# An Improved Quadrilateral Fitting Algorithm for the Water Column Contribution in Airborne Bathymetric Lidar Waveforms

**DOI:** 10.3390/s18020552

**Published:** 2018-02-11

**Authors:** Kai Ding, Qingquan Li, Jiasong Zhu, Chisheng Wang, Minglei Guan, Zhipeng Chen, Chao Yang, Yang Cui, Jianghai Liao

**Affiliations:** 1 Key Laboratory for Geo-Environment Monitoring of Coastal Zone of the National Administration of Surveying, Mapping and GeoInformation, Shenzhen University, Shenzhen 518060, China; dingkai@szu.edu.cn (K.D.); zhujiasong@gmail.com (J.Z.); guanminglei@szu.edu.cn (M.G.); chenzp@whu.edu.cn (Z.C.); yangchao161@sina.com (C.Y.); cuiyang@szu.edu.cn (Y.C.); liaojh@szu.edu.cn (J.L.); 2 Shenzhen Key Laboratory of Spatial Smart Sensing and Services, Shenzhen University, Shenzhen 518060, China

**Keywords:** LiDAR bathymetry, quadrilateral fitting, waveform processing, water column contribution

## Abstract

In this paper, an improved method based on a mixture of Gaussian and quadrilateral functions is presented to process airborne bathymetric LiDAR waveforms. In the presented method, the LiDAR waveform is fitted to a combination of three functions: one Gaussian function for the water surface contribution, another Gaussian function for the water bottom contribution, and a new quadrilateral function to fit the water column contribution. The proposed method was tested on a simulated dataset and a real dataset, with the focus being mainly on the performance of retrieving bottom response and water depths. We also investigated the influence of the parameter settings on the accuracy of the bathymetry estimates. The results demonstrate that the improved quadrilateral fitting algorithm shows a superior performance in terms of low RMSE and a high detection rate in the water depth and magnitude retrieval. What’s more, compared with the use of a triangular function or the existing quadrilateral function to fit the water column contribution, the presented method retrieved the least noise and the least number of unidentified waveforms, showed the best performance in fitting the return waveforms, and had consistent fitting goodness for all different water depths.

## 1. Introduction

Bathymetry is the measurement of underwater terrain, which is very important for a wide range of applications in hydrology, geomorphology, and meteorology. To date, several techniques for bathymetry retrieval have been developed for a variety of societal needs. The traditional bathymetry methods involve dropping a plumb line into the water or using a sonar system such as single-beam sonar, echo sounders, and multi-beam sounding [1]. However, these methods are labor-consuming, time-costly, expensive, and are limited to the navigable area. In contrast, rapid and inexpensive remote sensing methods, including radar, hyperspectral imagery, and photogrammetry, have been developed to estimate bathymetry [2]. These methods are better options than the in-situ measurement methods. However, radar and hyperspectral imagery cannot be used to directly retrieve water depth, and photogrammetry requires very clear water conditions [3]. Meanwhile, their precision is relatively poor, and they cannot provide a high-resolution distribution (errors in the order of 1 m) [4,5,6].

As an alternative, airborne light detection and ranging (LiDAR) bathymetry (ALB) has been developed as a powerful remote sensing technique that can be used to actively estimate bathymetry. By applying the ALB technique, the data can be obtained from waveform measurements of the received LiDAR pulse. This method can provide rapid operation with an acceptable accuracy and a high spatial density [7,8,9,10]. The ALB technique uses a pulsed blue-green laser as a bathymetric sensor that can penetrate the water to obtain the bathymetric data according to the differential arrival times of the laser echo signals reflected by the water surface and the sea bottom. In 1969, Hickman and Hogg first tested the performance of an airborne pulsed blue-green laser for near-shore bathymetric measurements [11]. Subsequently, a lot of ALB systems were built and tested in different areas in the 1970s. An operational prototype ALB system named LARSEN-500 was developed in Canada in 1985. This system used a Q-switched dual-band laser, operating simultaneously at 532 nm and 1064 nm [12]. Most of the early ALB systems were cumbersome to operate, and the costs were much higher than the other topographic systems. Moreover, a constant fraction discrimination (CFD) unit was used to retrieve the echo location by the airborne LiDAR scanner systems (ALSs), which only saves part of the time history of the returned LiDAR waveform.

With the rapid development of LiDAR and computer technologies, ALB systems are becoming smaller, lower cost, and more powerful. To date (as of December 2016), the latest ALB systems such as CZMIL-Nova, RIEGL VQ-880-G, Leica Chiroptera-II, and HawkEye-III have been widely applied for shallow-water surveying [13,14]. All these ALB systems have small-footprint full-waveform digitizing capabilities, which has resulted in great progress in bathymetry accuracy and target resolution. In general, a typical sample of a bathymetric LiDAR waveform is composed of surface return, bottom return, volume backscattering, and background noise. The bathymetry estimates can be retrieved from the LiDAR waveform by the use of a waveform processing algorithm. To date, several LiDAR waveform processing algorithms have been developed for the processing of full-waveform airborne LiDAR. These algorithms can be grouped into three main classes: (1) echo detection [15,16,17]; (2) mathematical approximation [18,19,20,21]; and (3) deconvolution methods [22,23,24,25,26]. In order to compare the performances of these different methods, Wang et al. [26] selected two algorithms for comparison in each class for single-wavelength bathymetric waveform processing, including peak detection (PD), the average square difference function (ASDF), Gaussian decomposition (GD), quadrilateral fitting (QF), Richardson-Lucy deconvolution (RLD), and Wiener filter deconvolution (WD). The results showed that the deconvolution methods excel in the accuracy of the water depth and the successful discovery rate, but their disadvantage is that they require the most time, due to the iterative step. The echo detection methods can obtain results in the shortest time, but their false discovery rate is higher than the other methods. The mathematical approximation methods require almost as much time as the deconvolution methods, and their accuracy is close to that of the echo detection methods. Based on continuous wavelet transform (CWT), Pan et al. [27] presented a novel full-waveform processing algorithm which is more stable than the Gaussian or empirical system response decomposition methods for shallow-river bathymetry [28]. From the comprehensive comparison of the different methods undertaken by Wang et al. [26], it was found that the mathematical approximation methods are less cost-efficient than the other methods.

However, by using the mathematical approximation methods, the LiDAR bathymetric waveform can be fitted as a combination of mathematical functions. These mathematical functions can represent the surface return, bottom return, and the water column response. In particular, the water column contribution records the relationship between amplitude and time, and based on this relationship, we can estimate the diffuse attenuation coefficient (*K_d_*) and the chlorophyll concentration profile, and it is also possible to classify the ocean water type [7,29]. Therefore, it is important to develop suitable algorithms to fit the water column contribution in LiDAR waveforms, which can not only improve the accuracy of bathymetry retrieval, but also obtain the optical properties and ecological parameters of water [30,31]. To date, several mathematical approximation methods have been used to fit LiDAR bathymetry full waveforms. In Long et al. [32], the water surface, water column, and water bottom contributions were approximated as a mixture of Gaussians. Abdallah et al. [19] separately fitted the water surface, the water column, and the bottom contributions as a Gaussian function, a triangular function, and a Weibull function, respectively. Abady et al. [20] developed a quadrilateral fitting algorithm, where two Gaussian functions were used to fit the surface and bottom contributions, and a quadrilateral function was used to fit the water column contribution. To sum up these mathematical approximation algorithms, it is relatively easy to fit the water surface and bottom returns, and the Gaussian function or Weibull function can show a satisfactory fit to the shape of the water surface or bottom contribution. However, the water column contribution is comparatively complicated, which is due to the fact that the water column contribution exhibits an asymmetric shape, physically corresponding to an infinite sum of successive translated Gaussian functions with an exponentially decaying amplitude along with water depth. Therefore, despite the fact that the above mathematical approximation algorithms are efficient, the fitting approaches cannot completely capture the physics of the interaction of the laser beam with the water column.

In this paper, we propose a new quadrilateral fitting algorithm to process bathymetric LiDAR waveforms and obtain bathymetry where: (1) Gaussian functions are used to fit both the surface and bottom components, and a new quadrilateral function is used to fit the water column contribution; (2) the new quadrilateral function is more in line with the fact that the water column response decreases exponentially with the increasing water depth; (3) the fittings are performed using a nonlinear least squares fitting approach according to the Levenberg-Marquardt algorithm [33]; and (4) we used a simulated dataset and a real dataset to test the presented waveform processing algorithm. The advantage of the new quadrilateral fitting algorithm we present was evaluated on sets of airborne simulated LiDAR waveforms and actual observed LiDAR waveform. In the following, for the simulated dataset, we compare the results of the bathymetry estimates (bias and standard deviation (STD)) obtained from the new quadrilateral function fitting method with those computed using a triangular function and the existing quadrilateral function initially presented by Abdallah et al. [19] and Abady et al. [19,20], and we discuss the sources of bias and deviation. What’s more, we investigate the influence of the parameter settings on the accuracy of the bathymetry estimates. For the real dataset, we compare the number of points with two returns recognized and the noise from all the algorithms. In addition, we assess the fitting results of return waveforms and compute the root mean squared error (RMSE) between the fitting and true values of the amplitude of return waveforms by using different algorithms.

## 2. Description of Datasets

### 2.1. Simulated Dataset

Due to the high cost of real data experiments, it is relatively expensive to collect ALB data. However, we can also validate the waveform processing algorithms using a simulated dataset based on the ALB laser propagation models. In general, the laser propagation models assume that the water column is homogeneous, with the same optical properties for the water column. These models can simulate full waveforms for different laser wavelengths in the optical spectrum from 300 nm to 1500 nm [26,34,35,36,37]. According to the existing laser propagation models, the return power can be expressed as:

(1) $$W_{R}\left(t\right)=W_{s}\left(t\right)+W_{c}\left(t\right)+W_{b}\left(t\right)+W_{n}\left(t\right)$$

where *t* is the time scale; *W_R_*(*t*) is the received waveform power; *W_s_*(*t*) is the power returned by surface reflection; *W_c_*(*t*) is the power returned by water column scatter; *W_b_*(*t*) is the power returned by bottom reflection; and *W_n_*(*t*) is the power of the noise, including the external noise from the air column and the internal noise from the detector.

The transmitted laser signal *W_T_*(*t*) is considered to be a time-delayed Gaussian distribution:

(2) $$W_{T}\left(t\right)=\frac{2}{T_{0}}\sqrt{\frac{\ln2}{\pi }}\exp\left(\frac{-4{\left(t-\tau \right)}^{2}\ln2}{T_{0}{}^{2}}\right)$$

where *T*_0_ is the full width at half maximum (FWHM) of the transmitted pulse, and *τ* is the delay of the laser group center with respect to the zero time.

The returned power from the water surface takes the form of:

(3) $$W_{s}\left(t\right)=\frac{P_{T}\eta \rho \cos^{2}\theta }{\pi H^{2}}\times W_{T}\left(t-\frac{2H}{v\cos\theta }\right)$$

where *P_T_* is the emitted power; *η* is the attenuation factor from the optical efficiency, atmosphere and receiver area; *ρ* is the interface reflectance; *θ* is the angle of incidence; *H* is the height of aircraft; *υ* is the speed of light in air. The interface reflectance *ρ* is defined by a Cook-Torrance geometric model with roughness of water surface (*r*), diffuse attenuation coefficient (*K_d_*), and angle of incidence (*θ*) as the input parameters [37,38]. 

The returned power from the water bottom is modeled as:

(4) $$W_{b}\left(t\right)=\frac{P_{T}\eta R_{b}F\exp(\frac{-2K_{d}D}{\cos\theta_{w}}){\left(1-\rho \right)}^{2}\cos^{2}\theta }{\pi {\left(nH+D\right)}^{2}}\times W_{T}\left(t-\frac{2H}{v\cos\theta }-\frac{2nD}{v\cos\theta_{w}}\right)$$

where *R_b_* is the reflectance of bottom; *F* denotes the loss coefficient based on the field of view effects; *θ_w_* denotes the angle of refraction; *D* denotes the water depth; and *n* denotes the refraction index.

The returned power from the water column is given by:

(5) $$W_{c}\left(t\right)=R_{c}(t)\times W_{T}\left(t\right)$$

where *R_c_*(*t*) is the water column response function, which is expressed as:

(6) $$R_{c}(t)=\left\{ \begin{array}{ll} \frac{P_{T}\eta \beta F\exp(\frac{-2K_{d}D}{\cos\theta_{w}}){\left(1-\rho \right)}^{2}\cos^{2}\theta }{{\left(nH+d(t)\right)}^{2}} & 0\leq d(t)\leq D\\ 0 & other \end{array}\right.$$

where *β* denotes the volume scattering function; *d*(*t*) denotes the column layer; and *K_d_* denotes the diffuse attenuation coefficient.

The noise is defined as Gaussian white noise with a constant STD *σ_n_* and zero mean. The noise is determined by the peak signal-to-noise ratio (PSNR) [39]:

(7) $$\sigma_{N}=\frac{Max\left(W_{s}\left(t\right)+W_{c}\left(t\right)+W_{b}\left(t\right)\right)}{PSNR}$$

In the simulated dataset, we set both the fixed parameters and the floating parameters. For the fixed parameters, we used the usual green wavelength (*λ*: 532 nm) common to ALB systems, the FWHM of the transmitted pulse (*T*_0_: 7 ns), the transmitted power (*P_T_*: 0.5 mW), the aircraft altitude (*H*: 200 m), the backscattering coefficient (*β*: 4 × 10^−4^ m^−1^sr^−1^), the loss coefficient due to the field of view effect (*F*: 1), the two-way time delay (*τ*: 0 ns), and the light velocity in air (*v*: 3 × 10^8^ m/s), and the attenuation factor from the optical efficiency (*η*: 0.01). For the floating parameters, we set the diffuse attenuation coefficient (*K_d_*), the bottom reflectance (*R_b_*), the roughness of water surface (*r*), the incidence angle (*θ*), the depth of water (*D*), and the PSNR to be varying, so that we could test the algorithm performance in a variety of conditions. These parameters were set as listed in Table 1.

**Figure 1:**
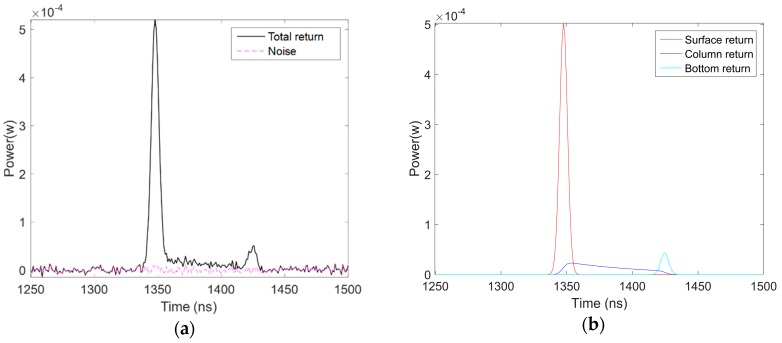
An example of a simulated return waveform using airborne sensor parameters: (**a**) a return waveform with noise; and (**b**) the return waveform without noise, which is decomposed into surface return, column return, and bottom return.

Based on the settings in Table 1, the simulated waveforms were generated with random water properties and sensor parameters at a given water depth. An example of a simulated waveform is depicted in Figure 1:

### 2.2. Real Dataset

The real airborne LiDAR bathymetric data was collected from Aquarius Airborne Laser Terrain Mapper (ALTM) system. It is a single wavelength shallow water bathymetry sensor developed by Teledyne Optech Inc. (Toronto, ON, Canada) and operated by the National Center for Airborne Laser Mapping (Houston, TX, USA). The Aquarius system is a new single-wavelength airborne LiDAR bathymetry system developed in 2011 for shallow-water bathymetry with high-resolution and light weight units. This system is capable to take measurements of simultaneous land and shallow water depth. It employs a frequency-doubled ND: YAG laser with a single-wavelength of 532 nm (green), a half-swath angle 20°, a 33 KHz pulse repetition frequency, and 1 mrad beam divergence that yields a laser footprint one 1000th of the laser optical range, the main technical characteristics of Aquarius system in shallow water mode was shown in Table 2 [40].

The experimental dataset was captured at an altitude of 300 m by the Aquarius system at Wuzhizhou Island, Sanya, Hainan, Southern China, on 19 December 2012. In this paper, a part of the dataset over a coastal area (about 240 × 80 m) was used to assess the proposed waveform processing algorithm. The dataset consisted of waveform data, discrete data, and image data. The waveform was recorded with waveform digitizer and the CFD is applied on the analog signal to detect the effective returns. The received full waveform data for each shot and the corresponding GPS times are recorded by a 12 bit Intelligent Waveform Digitizer (IWD-2). The discrete data were retrieved by the Aquarius CFD, and processed into las format by use of Optech software. The image data were optical photos taken by a DiMAC ULTRA-LIGHT+ aerial camera system (DiMAC, Charleroi, Belgium). 

The Aquarius waveform data consists of time-series amplitude data of numerous laser shots. For each laser shot, both the transmitted and returned amplitude waveform are recorded. To organize the large number of laser shots, a certain amount of sequential shots are grouped into a frame. Each frame has a unique frame number and a GPS time. The GPS time is defined as the acquired time for the first shot in the frame. With the GPS time of frames and the number of shots in each frame, the interval time between two neighboring laser shots can be calculated. Correspondingly, the GPS time of each shot in the frame can be determined. The GPS time for the shots are then used to synchronize with positioning data collected by the ALTM.

## 3. Methods

### 3.1. Mathematical Approximation Method

As a widely used strategy in the processing of LiDAR waveforms, mathematical approximation algorithms are commonly used to fit the received waveform by the use of a combination of mathematical functions, such as a Gaussian function, lognormal function, and Weibull function [18]. The most commonly used algorithms are the triangular fitting algorithm and the quadrilateral fitting algorithm.

#### 3.1.1. Triangular Fitting Algorithm

The triangular fitting algorithm (TF) is normally used to decompose a laser altimeter return waveform into a series of functions: Gaussian functions for both the water surface and water bottom components, and a triangular function to fit the water column component.

**Figure 2:**
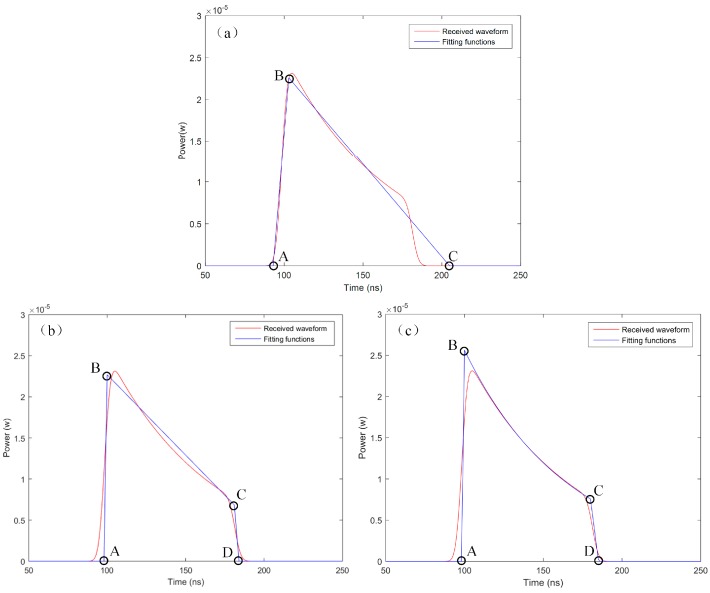
Fitting examples of the column contribution for a simulated waveform without noise, using the airborne sensor parameters: (**a**) triangular fitting function; (**b**) quadrilateral fitting function; and (**c**) improved quadrilateral function.

The Gaussian function is as follows:

(8) $$f(t)=\sum_{i=1}^{N}\alpha_{i}e^{-\frac{{\left(t-\mu_{i}\right)}^{2}}{2\delta_{i}{}^{2}}}$$

where *N* is the number of Gaussian components, which can be selected through an iterative process [41,42]. $\alpha_{i}$, $\mu_{i}$ and $\delta_{i}$ are the amplitude, location, and half-width of the ith Gaussian component. However, since a bathymetric LiDAR waveform is mainly composed of a water surface return, a bottom return, and a water column return, we adopt the approach used in a previous study of using two Gaussian functions (*N* = 2) to fit the received waveform [43]. A triangular function is used to fit the water column contribution, and the function ($f_{T}(t)$) is expressed as:

(9) $$f_{T}(t)=\left\{\begin{array}{ll} 0 & t\leq a\\ d\times \left(\frac{t-a}{b-a}\right) & a<t\leq b\\ d\times \left(\frac{t-c}{b-c}\right) & b<t\leq c\\ 0 & t>c \end{array}\right\}$$

where *a*, *b*, and *c* are the locations of three edge points (A, B and C) of the triangle, and *d* is the magnitude for the second point (B). An example of triangular fitting is shown in Figure 2a.

The parameters for the two Gaussian functions and the triangular function can be retrieved by minimizing the following cost function:

(10) $$f_{c}(t)=\frac{1}{N}\left\|W_{R}\left(t\right)-\sum_{i=1}^{2}\alpha_{i}e^{-\frac{{\left(t-\mu_{i}\right)}^{2}}{2\delta_{i}{}^{2}}}-f_{T}(t)\right\|$$

We use nonlinear least squares fitting according to the Levenberg-Marquardt algorithm to optimize the cost function [33]. In addition, we set the initial parameters for the Gaussian functions so as to get rid of the meaningless results based on the detected target responses by the use of a PD method [27]. The following algorithms also follow these steps.

#### 3.1.2. Quadrilateral Fitting Algorithm

The quadrilateral fitting algorithm (QF) uses a quadrilateral function to fit the water column contribution. The cost function is defined as shown in Equation (11), and the quadrilateral function can be expressed as shown in Equation (12):

(11) $$f_{c}(t)=\frac{1}{N}\left\|W_{R}\left(t\right)-\sum_{i=1}^{2}\alpha_{i}e^{-\frac{{\left(t-\mu_{i}\right)}^{2}}{2\delta_{i}{}^{2}}}-f_{q}(t)\right\|$$

(12) $$f_{q}(t)=\left\{\begin{array}{ll} 0 & t\leq a\\ e\times \left(\frac{t-a}{b-a}\right) & a<t\leq b\\ \frac{ec-bg+t(g-e)}{c-b} & b<t\leq c\\ g\times \left(\frac{t-d}{c-d}\right) & c<t\leq d\\ 0 & t>d \end{array}\right\}$$

where, as shown in Figure 2b, A(*a*, 0), B(*b*, *e*), C(*c*, *g*), and D(*d*, 0) are, respectively, the four edge points of the quadrilateral; *a*, *b*, *c*, and *d* are the locations of the four edge points; and *e* and *g* are the magnitudes for points B and C.

#### 3.1.3. Improved Quadrilateral Fitting Algorithm

Based on the above-described quadrilateral fitting algorithm, we propose a new quadrilateral function to fit the water column contribution. Firstly, according to the relationship between the laser amplitude and time in the water column, we construct an exponential function f(t), which is given by:

(13) $$f\left(t\right)=m\times \alpha^{t}$$

where *m* and α are undetermined coefficients, and we first assume that the curve f(t) passes through two points B(*b*, *e*) and C(*c*, *g*):

(14) $$\left\{ \begin{array}{l} e=m\times \alpha^{b}\\ g=m\times \alpha^{c} \end{array}\right.$$

(15) $$\frac{g}{e}=\alpha^{c-b}$$

Secondly, we take the natural logarithm of both sides of Equation (15):

(16) $$\ln\frac{g}{e}=(c-b)\ln\alpha$$

We then rearrange the equation and obtain:

(17) $$\alpha =\exp(\frac{\ln g-\ln e}{c-b})$$

Substituting Equation (17) into Equation (14), we obtain:

(18) $$m=\exp(\frac{c\ln e-b\ln g}{c-b})$$

Next, substituting Equations (17) and (18) into Equation (13), we obtain the following equation after rearranging:

(19) $$f(t)=\exp(\frac{(\ln g-\ln e)t+c\ln e-b\ln g}{c-b})$$

Finally, the new quadrilateral fitting function can be expressed as:

(20) $$f_{q}(t)=\left\{\begin{array}{ll} 0 & t\leq a\\ e\times \left(\frac{t-a}{b-a}\right) & a<t\leq b\\ \exp(\frac{(\ln g-\ln e)t+c\ln e-b\ln g}{c-b}) & b<t\leq c\\ g\times \left(\frac{d-t}{d-c}\right) & c<t\leq d\\ 0 & t>d \end{array}\right\}$$

An example of the improved quadrilateral function to fit the water column contribution is shown in Figure 2c.

Figure 2 demonstrates that the three fittings are applied to the same simulated waveform without noise, using the airborne sensor parameters, which shows the efficient fitting in this schematic case. Comparing Figure 2a–c, we can see that the quadrilateral function performs a better fitting to the shape of the water column component than the triangular function. Furthermore, the improved quadrilateral function shows a better fitting to the shape of the water column than the existing quadrilateral function. What are the causes of the different fitting results and performances? Can the improved quadrilateral algorithm improve the performance in extracting the water depth over the existing quadrilateral algorithm? How do the water conditions and parameter settings of the ALB system affect the bathymetry accuracy? To answer these questions, a simulated dataset obtained by the use of the laser propagation model (Section 2.1) was used to validate the improved quadrilateral fitting algorithm (IQF). The metrics used in the algorithm evaluation and comparison are given in the next section.

### 3.2. Methodology for the Comparison

To verify the superiority of the proposed algorithm, a comparative experiment was designed as follows:

Firstly, we compared the results of the water depths retrieved by the use of each algorithm. Because we knew the true values of the water depths from the simulated waveform dataset, the estimated values could be compared with the true values using the metrics, which are defined as follows:

(1) The success rate, which is defined as the percentage of successfully detected points (points with ≥2 returns detected and errors of less than 1 m):
  (21) $$S_{r}=\frac{\#\mathrm{of}\;\mathrm{successfully}\;\mathrm{detected}\;\mathrm{points}}{\mathrm{of}\;\mathrm{all}\;\mathrm{the}\;\mathrm{points}}$$
(2) The false discovery rate, which is defined as the percentage of wrongly detected points (points with ≥2 returns detected and errors larger than 1 m):
  (22) $$F_{r}=\frac{\#\mathrm{of}\;\mathrm{wrongly}\;\mathrm{detected}\;\mathrm{points}}{\mathrm{of}\;\mathrm{all}\;\mathrm{the}\;\mathrm{points}}$$
(3) The bias, which is defined as the difference between an estimated expected value and the true value of the parameter being estimated, where ${D^{\prime }}_{i}$ and $D_{i}$ are the estimated and true water depths for the *i*th case, and *N* is the number of forward modeling cases:
  (23) $$Bias=\frac{\sum_{i}^{N}\left({D^{\prime }}_{i}-D_{i}\right)}{N}$$
(4) The STD is used to quantify the amount of variation or dispersion of a set of data values, where ${\bar{D}}^{\prime }$ is the mean of the estimated water depths ($D^{\prime }$):
  (24) $$STD=\sqrt{\frac{\sum_{i}^{N}{\left({D^{\prime }}_{i}-{\bar{D}}^{\prime }\right)}^{2}}{N}}$$
(5) The root-mean-square error (RMSE) between the true and estimated values of the depth of water under different parameter settings:
  (25) $$RMSE=\sqrt{\frac{\sum_{i}^{N}{\left({D^{\prime }}_{i}-D_{i}\right)}^{2}}{N}}$$
(6) R-squared (*R*^2^), to estimate the fitness of the true depths of the successfully detected points, where *NS* is the number of successfully detected points, and $\bar{D}$ is the mean of the true water depths (*D*):
  (26) $$R^{2}=1-\frac{\sum_{i}^{NS}{\left({D^{\prime }}_{i}-D_{i}\right)}^{2}}{\sum_{i}^{NS}{\left(D_{i}-\bar{D}\right)}^{2}}$$
(7) The time cost (T), to evaluate the efficiency of the algorithm. We adopted parallel computing (eight CPU cores used simultaneously) to accelerate the computations in this paper.

Secondly, the robustness of each algorithm was tested by the use of a Monte Carlo method. We then examined the influence of the parameter settings on all the algorithms. The six floating parameters shown in Table 1 were tested: PSNR, bottom reflectance (*R_b_*), surface roughness (*r*), diffuse attenuation coefficient (*K_d_*), water depth (*D*), and scan angle (*θ*). The range for each parameter was divided equally into 50 bins. The depth RMSEs for the full waveforms inside each bin were computed. The RMSEs for the function of each parameter were plotted to study the influence of the parameter settings.

## 4. Results and Discussion

### 4.1. Simulated Dataset

#### 4.1.1. Performance Assessments

Firstly, we ran the above-mentioned laser propagation model to acquire one million simulated waveforms. The parameters for each waveform were defined in Table 1.

Secondly, the three algorithms were used to process these waveforms, respectively. The performances of the three algorithms with the simulated dataset are shown in Table 3.

The above table demonstrates that the improved quadrilateral fitting algorithm shows a higher success rate than the other two algorithms. The false discovery rate for the improved quadrilateral fitting algorithm is lower than that of the other two algorithms. The improved quadrilateral fitting algorithm shows the best performance (the lowest RMSE_D_) in water depth retrieval. The bias of the improved quadrilateral fitting algorithm is improved by 0.269 m and 0.092 m compared with the triangular fitting algorithm and the previous quadrilateral fitting algorithm, respectively. When comparing the STD, it can be seen that the improved quadrilateral fitting algorithm performs the best, and the second-best algorithm is the existing quadrilateral fitting algorithm. The R-squared values show that the improved quadrilateral fitting algorithm is best able to fit the true depths of the successfully detected points, and they also suggest that the test algorithms do not differ too much in their ability to process the successfully detected points. The time cost for the improved quadrilateral fitting algorithm is much higher than for the other methods. Conversely, the triangular fitting algorithm shows the best computational efficiency.

#### 4.1.2. Accuracy Calculations

In this paper, as a metric, the RMSE is combined with the bias and STD. Therefore, the two elements for different water depths (1–15 m) were further calculated by Equations 23,24, respectively. The relationship between RMSE, STD, and bias is described as follows:

(27) $${\mathrm{RMSE}}^{2}{=\mathrm{STD}}^{2}{+\;\mathrm{Bias}}^{2}$$

**Figure 3:**
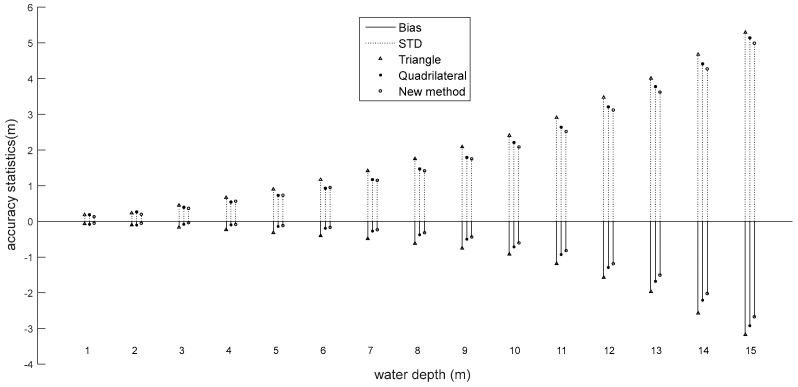
Bias and STD of the bathymetry estimates versus water depth.

**Figure 4:**
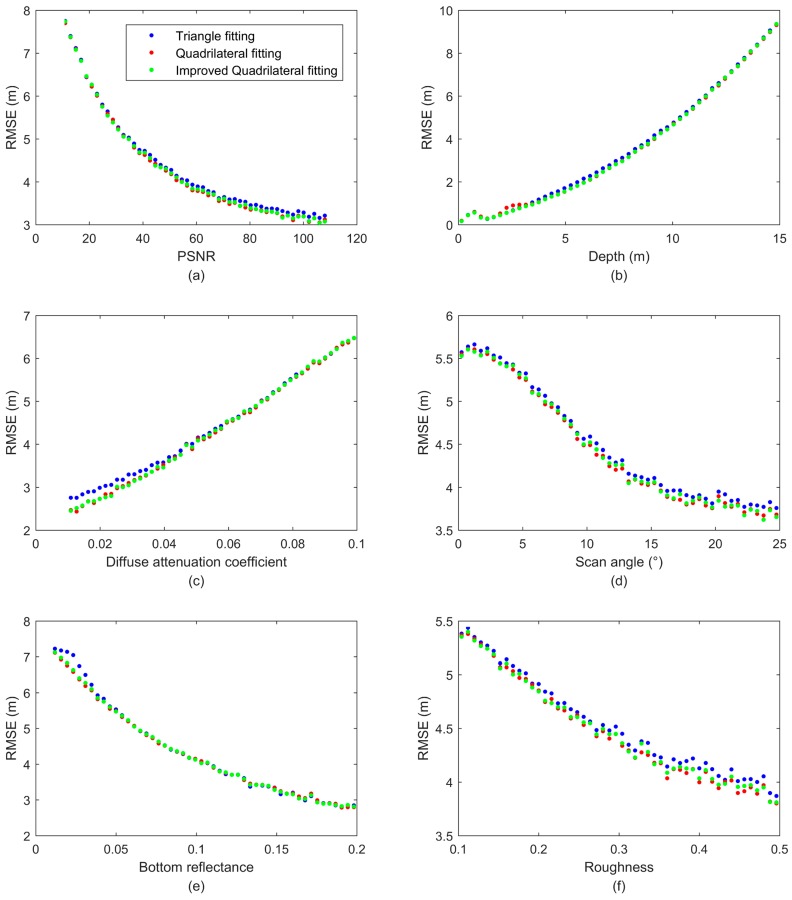
The RMSE changes in the function of one parameter: (**a**) PSNR; (**b**) Depth; (**c**) Diffuse attenuation coefficient; (**d**) Scan angle; (**e**) Bottom reflectance, and (**f**) Roughness.

The STD and bias make a similar contribution to the RMSE. Comparing Figure 3 and Figure 4, we can see that the trends of STD and bias are in agreement with the RMSE. Apart from the very shallow depths, all the metrics increase with the increasing water depth. In very shallow water, the detection error is larger than when the surface and bottom returns are intermingled. The overall bias and STD for all the water depths are shown in Table 4.

As depicted in Figure 3, the bias at each water depth for the simulated dataset shows an obvious growth with the increasing water depth, ranging from −0.046 m at a water depth of 1 m to −2.679 m at a water depth of 15 m for the improved quadrilateral fitting algorithm, which is better than the −0.0819 m at a water depth of 1 m to −2.923 m at a water depth of 15 m for the existing quadrilateral fitting algorithm, and −0.068 m at a water depth of 1 m to −3.174 m at a water depth of 15 m for the triangular fitting algorithm. Moreover, the overall bias shows an improvement of approximately 0.087 m and 0.284 m in magnitude (See Table 4) when using the improved quadrilateral fitting procedure compared with the existing quadrilateral fitting algorithm and the triangular fitting algorithm. The bias increases as the water depth increases, which is due to the bottom response decreasing as the water depth increases.

Similarly, the STD also greatly increases as the water depth increases, ranging from 0.139 m to 4.989 m when using the improved quadrilateral fitting algorithm, 0.186 m to 5.140 m when using the existing quadrilateral fitting algorithm, and 0.186 m to 5.286 m when using the triangular fitting algorithm. The overall STD for all the water depths is shown in Table 4, which clearly displays the improvement in reducing the STD by 0.064 m and 0.248 m in magnitude when using the improved quadrilateral fitting algorithm compared with the existing quadrilateral fitting algorithm and the triangular fitting algorithm, respectively.

As depicted in Equation 4, the bottom return decreases with the increasing water depth. Therefore, it can also be seen that the bias shows an exponential increase with the water depth (see Figure 3). The capability of the different algorithms, as revealed by the RMSE, is consistent with the results using the STD and bias. Compared with the other two algorithms, the improved quadrilateral fitting algorithm obtains the smallest STD and bias values. However, the difference in the bias between algorithms is more distinct than the STD, implying that the main way we could improve the performance of the quadrilateral fitting algorithm is by reducing the bias. According to recent research, the bias mainly comes from the overlaps of the water column component on the water surface and bottom returns, and the bottom geometry can also generate a bottom peak shift; however, we did not model the bottom geometry, so the shift could not be explained. The STD mainly comes from the variability of the water column parameters and random noise [20,26,44]. Therefore, the experimental results suggest that a better algorithm to fit the water column component could play an important role in improving the accuracy of bathymetry estimation.

#### 4.1.3. RMSE Changes in the Function of One Parameter

To understand the capabilities of the improved quadrilateral fitting algorithm, which shows a better performance than the existing quadrilateral fitting algorithm and the triangular fitting algorithm in retrieving both the water magnitude and depth, we further studied the influence of the parameter settings on the three algorithms and tested the robustness of each algorithm by using a Monte Carlo method. We plotted the RMSEs in the function of each parameter (see Figure 4). We can see that all the parameters clearly affect the RMSE [45]. It can be seen that the RMSE displays an exponential decay with the increasing PSNR, implying that the accuracy of the water depth extraction is greatly affected by the noise. However, when the PSNR reaches 50, the downward trend is slow. This illustrates that a PSNR of 50 would be enough to retrieve the water depth from a LiDAR waveform. And we can find that the proposed algorithm has the least averaged RMSE values for different PSNR distribution (0–40 40–80, and 80–120), as shown in Table 5. It can also be seen that the water depth greatly affects the accuracy of the water depth retrieval. When the water depth varies from 0 to 15 m, the RMSE shows an exponential increase with the increasing water depth. Such a result is reasonable because the water depth plays an important role in obtaining the bottom return power. According to Equation (4), the laser power displays an exponential decay with the increase of water depth. Therefore, a strong bottom return will decrease the difficulty in retrieving water depths. However, it can be seen that the RMSE is affected when the water depth is too small (<1 m), which implies that the surface and bottom returns are mixed together at a very shallow water depth, so it is very difficult to distinguish the two peaks from the returns. A weak bottom reflectance can produce a weak bottom signal; therefore, the RMSE exhibits an exponential decrease with the increasing bottom reflectance. A large diffuse attenuation coefficient can result in a weak bottom signal, which brings about a large RMSE. The scan angle and roughness mainly affect the surface return, which can be easily detected by the ALB system; therefore, the two parameters have less of an influence than the other parameters. Nevertheless, the surface reflection is still associated with the bottom response, which is due to the fact that if less laser power is reflected by the water surface, more laser power will penetrate the water. Based on the Cook-Torrance geometric optics model, the surface reflection is affected by the scan angle and roughness in a nonmonotonic method [38]. However, as shown in Figure 4, it can also be seen that the RMSE indicates a decreasing trend with the increasing scan angle and roughness. Such a result can be explained by the fact that the surface reflection decreases with the increasing scan angle and roughness, which leads to the larger bottom response. The curves of the diffuse attenuation coefficient and bottom reflectance for the three algorithms show a similar form; therefore, we can choose the bathymetric LiDAR waveform processing algorithm without considering the values of these parameters.

### 4.2. Real Dataset

**Figure 5:**
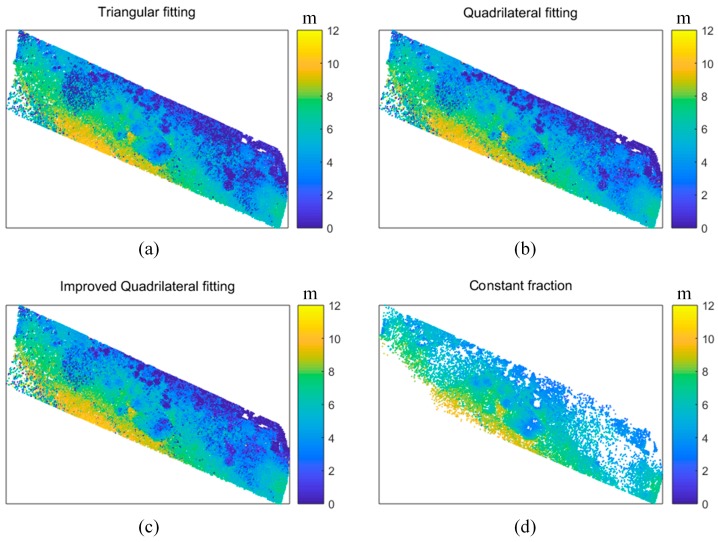
Bathymetry maps from all the algorithms: (**a**) Triangular fitting algorithm; (**b**) Quadrilateral fitting algorithm; (**c**) Improved quadrilateral fitting algorithm, and (**d**) CFD from the Aquarius data.

To further verify the superiority of the proposed algorithm, a real dataset (introduced in Section 2.2) was used to assess the algorithms. Figure 5 shows the bathymetry derived from the three algorithms and the CFD from the Optech Aquarius data. The points with two returns recognized are displayed (depth > 0). It can be seen that all the three algorithms obtained more points than the CFD, indicating that the more detailed information can be retrieved by use of the waveform processing algorithm. The triangular fitting algorithm gave the second lowest number of points, in contrast, the improved quadrilateral fitting algorithm gave the most number of points. In addition, it can be found that some salt-and-pepper noise distributed in the bottom-left, bottom-middle and top-left of the bathymetry maps (see Figure 5a–c). Because the underwater bottom topography is spatially smooth, we can conclude that the noise should be the errors from the algorithms. Comparing the three bathymetry maps carefully (Figure 5a–c), we can find that the results from the improved quadrilateral fitting algorithm is relatively less noisy than for the other two algorithms.

**Figure 6:**
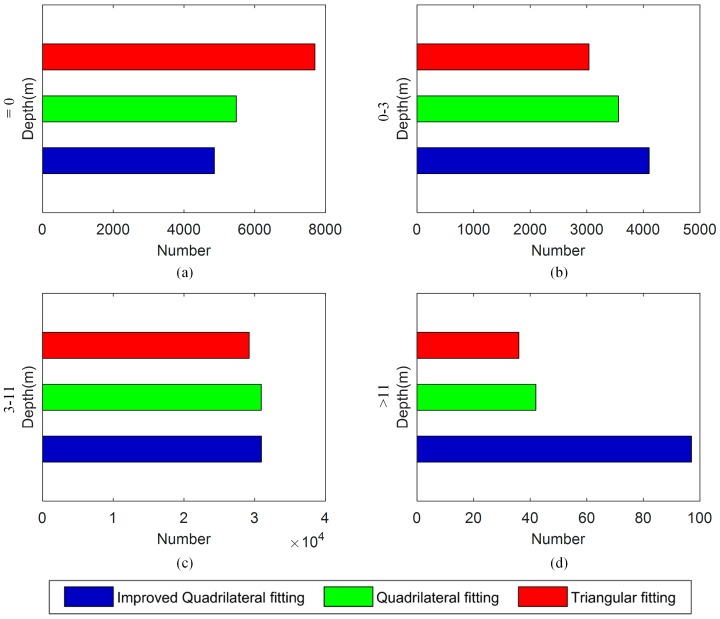
The bathymetry distribution of the detected waveform numbers for the three algorithms: (**a**) Depth = 0 (m); (**b**) 0 < Depth ≤ 3 (m); (**c**) 3 < Depth ≤ 11 (m), and (**d**) Depth > 11 (m).

To further compare the algorithms, the water depths distribution of the recognized waveform number was computed, as shown in Figure 6. It was found that the improved quadrilateral fitting algorithm retrieved the least number of unidentified waveforms (depths = 0). And in the very shallow water (0 < depths < 3 m), higher detection rates were also found in the improved quadrilateral fitting algorithm than the other two algorithms. Over the shallow-medium water depth (3 < depths < 11 m), the two quadrilateral fitting algorithms retrieved similar detection numbers, where the improved quadrilateral fitting algorithm performed slightly higher detection rates than the existing quadrilateral fitting algorithm. In the deep water depth (depths > 11 m), it can be seen obviously that the improved quadrilateral fitting algorithm obtained a much greater number of detected points than the other waveform processing algorithms. The results mean that the improved quadrilateral fitting algorithm performed better performance in the very shallow water and deep water. The simulated experiment in Section 4.1 also showed that the improved quadrilateral fitting algorithm had a high successful rate from which we can deduce that the bathymetry detected by the improved algorithm is reliable. Next, we randomly selected some waveforms detected by the existing quadrilateral fitting algorithm or the improved quadrilateral fitting algorithm, separately.

**Figure 7:**
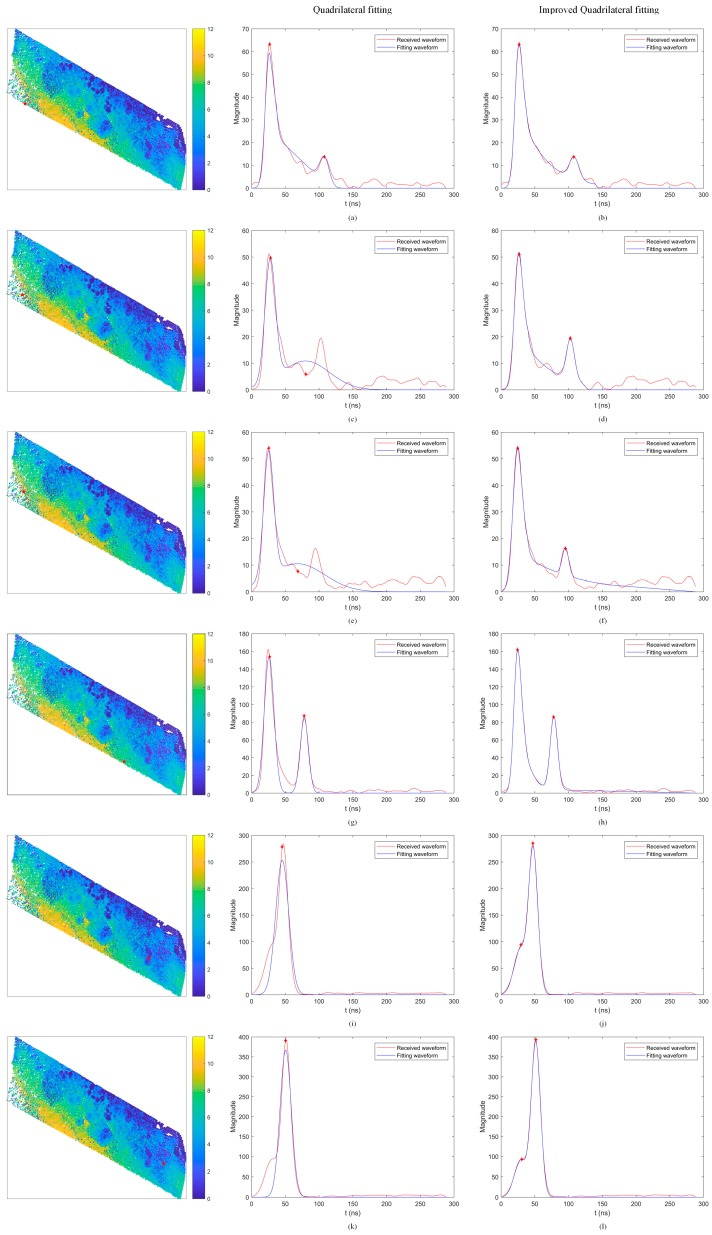
Six randomly selected waveforms detected solely by the QF and IQF algorithms. (**a**,**c**,**e**,**g**,**i**,**k**): QF algorithm; (**b**,**d**,**f**,**h**,**j**,**l**): IQF algorithm.

The waveforms and their detected surface and bottom locations were displayed in Figure 7. It can be found that the improved quadrilateral fitting shows a better fitting to the waveforms than the existing quadrilateral fitting. Especially when the change of the amplitude along with water depth is significant in the water column return, the water column and bottom return cannot be fitted well, which results in an obvious error in the bottom location (see Figure 7c,e). In contrast, the water column and bottom return can be fitted well, therefore their detected bottom locations can be retrieved easily (see Figure 7d,f). It can also be seen that the water column component cannot be obtained by using the existing quadrilateral fitting algorithm in some cases (see Figure 7g), however, when using the improved quadrilateral fitting algorithm, we can obtain the water column component (see Figure 7h). In general, we can find that when the shape of return waveforms is fat, which could be produced by a combination of two adjacent returning pulses, only a location can be detected by using the existing quadrilateral fitting algorithm (see Figure 7i,k), in contrast, the waveforms can be recognized by using the improved quadrilateral fitting algorithm (see Figure 7j,l).

To quantitatively compare the fitting results, we calculated the root mean squared error (RMSE) between the fitting and true values of the amplitude of return waveforms by using different algorithms. We used $RMSE=\sqrt{\frac{\sum_{i=1}^{n}(y_{i}-{y^{\prime }}_{i}{)}^{2}}{n}}$ to measure the *RMSE*, where *y* is the amplitude of true return waveform, *y*′ is the amplitude of the fitting return waveform. Finally, we obtained the averaged *RMSE* of all the return waveforms, the results are shown in Table 6. We can see that the improved quadrilateral algorithm showed the best performance (the least RMSE) in fitting the return waveforms.

**Figure 8:**
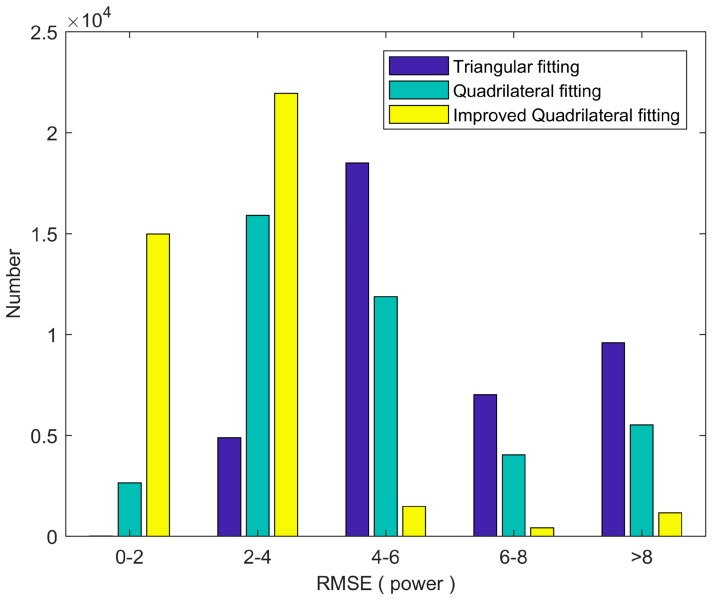
The RMSE distribution of the detected waveform numbers for the three algorithms.

**Figure 9:**
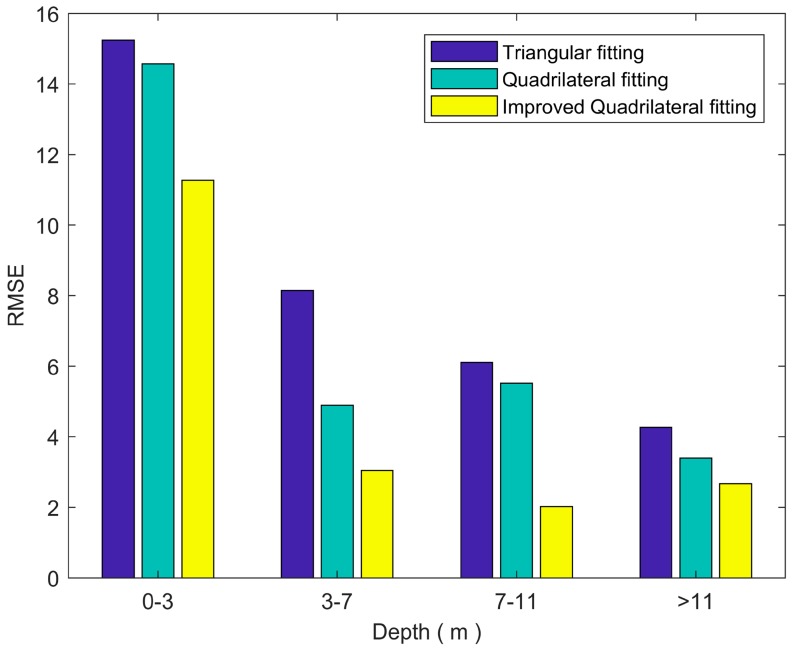
The depth distribution of the RMSE value for the three algorithms.

To further compare the three algorithms, we calculated the RMSE (power) distribution of the detected waveform number, as shown in Figure 8. It can be found that the improved quadrilateral fitting algorithm retrieved the most number with low RMSE (RMSE < 4), and the least number with high RMSE (RMSE > 4). This is similar to the simulated data, where the improved quadrilateral fitting algorithm obtained the lowest RMSE_D_ in water depth retrieval. As seen in Figure 9, we found that the proposed waveform fitting algorithm has consistent fitting goodness for all different water depths.

To sum up, as seen in the simulated and real experiments, the quadrilateral fitting algorithm shows a satisfactory performance when compared with the triangular fitting algorithm. Such a result is reasonable because the use of the quadrilateral function to fit the water column return can obtain a much better shape than the triangular function. A previous study also suggested that the quadrilateral fitting algorithm can bring about a better performance than the triangular fitting algorithm in the accuracy of the water depth extraction [20]. However, the quadrilateral fitting algorithm requires a much longer time than the triangular fitting algorithm, because the former has more parameters than the latter. It can also be seen that the improved quadrilateral fitting algorithm requires a longer time than the existing quadrilateral fitting algorithm. Such a result is reasonable in that curve fitting requires much more time than linear fitting. When comparing all the metrics except for the time cost, we can see that the improved quadrilateral fitting algorithm performs better than the existing quadrilateral fitting algorithm. Such a result can be explained by the fact that when the laser penetrates the water surface, the received signal power exhibits an approximately exponential decay with the increase of water depth. For the improved quadrilateral fitting algorithm, the quadrilateral function follows the transmitting process of decay; however, for the existing quadrilateral fitting algorithm, the received signal power exhibits an approximately linear decay with the increase of water depth. In bathymetric LiDAR, the column contribution is a time-dependent function and cannot be simply modeled as a linear function. Therefore, we are able to conclude that the improved quadrilateral fitting algorithm is effective and feasible in practical use.

## 5. Conclusions

In this study, an improved quadrilateral fitting algorithm to process bathymetric LiDAR waveforms was tested on a simulated dataset obtained from the laser propagation model and a real dataset from Optech Aquarius system. The other two common waveform processing algorithms were adopted for a comparison with the new algorithm for single-waveform bathymetric LiDAR. The main conclusions are as follows:

(1) The new fitting algorithm we presented shows an improvement over the water depth retrieved by the triangular fitting algorithm and the existing quadrilateral fitting algorithm, however, its disadvantage is that it costs the most time, due to its nonlinear curve fitting. The triangular fitting algorithm requires the least time, but its disadvantage is that the number of detected waveforms is less than for the other two algorithms, and it obtains a relatively high RMSE_D_ for the retrieved water depth and a higher false discovery rate. Through experiments by using simulated dataset and real dataset, we can find that the new quadrilateral function shows a better fit to the shape of the water column return than the existing quadrilateral function. Therefore, it’s much easier to get the surface and bottom locations by using the improved quadrilateral fitting algorithm.
(2) For the simulated dataset, using the improved quadrilateral function, the results show an improvement of 0.269 m and 0.092 m in bias compared with the triangular fitting algorithm and the existing quadrilateral fitting algorithm. In addition, the overall STD shows an improvement of 0.282 m and 0.123 m when using the improved quadrilateral fitting procedure compared with the triangular fitting algorithm and the existing quadrilateral fitting algorithm. For the real dataset, comparing the other two algorithms, the improved quadrilateral fitting algorithm retrieved the least noise and the least number of unidentified waveforms, especially performed better performance in very shallow water (0–3 m) and deep water (>11 m). What’s more, the improved quadrilateral algorithm showed the best performance (the least RMSE (power)) in fitting the return waveforms, and had consistent fitting goodness for all different water depths.
(3) The mathematical approximation algorithms mainly depend on the step of cost function optimization, which may generate abnormal values or run into local minima. Moreover, the diffuse attenuation coefficient, bottom reflectance, noise level, and water depth play important roles in the RMSE of the bathymetry estimation. The performance of the algorithms is only slightly affected by the roughness of the water surface and scan angle, but it is greatly impacted in very shallow water. Nevertheless, the algorithms perform relatively well under certain conditions when having smaller diffuse attenuation coefficients, higher bottom reflectance, lower noise levels, and shallower water. Therefore, the choice of algorithm not only depends on the performance of the algorithm, but also depends on the actual application.

## Figures and Tables

**Table 1:** Main parameter settings for the simulated dataset.

Fixed Parameters	Values	Floating Parameters	Values
*λ*(nm)	532	*K_d_* (m^−1^)	0.01–0.1
*T*_0_ (ns)	7	*R_b_*	0.01–0.2
*P_T_* (W)	5.0 × 10^−4^	*r*	0.1–0.5
*H* (m)	200	*θ* (°)	0–25
*Β* (m^−1^sr^−1^)	4.0 × 10^−4^	*D* (m)	0–15
*F*	1	PSNR	10–110
*Τ* (ns)	0		
*v* (m/s)	3.0 × 10^8^		
*η*	0.01		

**Table 2:** Main technical characteristics of Aquarius in shallow water mode.

Parameter	Specification
Flight height (AGL, m)	300–600
Laser wavelength (nm)	532
Power	28 V; 900 W; 35 A (peak)
Pulse width (FWHM in ns)	8.3
Digitization frequency (GHz)	1
Resolution of full waveform (bits)	12
Beam divergence (mrad)	1
Pulse repetition rate (KHz)	33, 50, 70
Scan rate (Hz)	0~70
Scan half-angle	0~±25°
Point density (pts/m^2^)	4
Footprint on water surface (cm)	30~60
Depth range (m)	0~ > 10 (for *K_d_* < 0.1 m^−1^)

**Table 3:** Performance assessments of the three mathematical approximation algorithms for waveform processing.

Algorithm	Sr (%)	Fr (%)	RMSE_D_ (m)	Bias (m)	STD (m)	R^2^	Tc (s)
TF	73.25	8.6362	2.6377	−0.8299	2.5037	0.9733	1804.1613
QF	75.17	6.1840	2.4337	−0.6530	2.3444	0.9786	2350.6966
IQF	75.68	5.6471	2.2910	−0.5607	2.2213	0.9837	4627.2592

**Table 4:** Overall bathymetry accuracy (bias and STD) using the simulated dataset.

	TF	QF	IQF
Bias (m)	−0.970	−0.773	−0.686
STD (m)	2.107	1.924	1.859

**Table 5:** The PSNR distribution of the averaged RMSE values for the three algorithms.

PSNR	RMSE (m)	RMSE (m)	RMSE (m)
	TF	QF	IQF
0–40	5.601	5.551	5.545
40–80	3.358	3.225	3.213
80–120	2.684	2.509	2.483

**Table 6:** Averaged RMSE of the return waveforms using the Aquarius dataset.

Algorithms	TF	QF	IQF
Averaged RMSE (power)	7.586	5.158	2.775
